# Polycystic Ovarian Syndrome in Adolescents

**DOI:** 10.7759/cureus.34183

**Published:** 2023-01-25

**Authors:** Avanti Adone, Darshna G Fulmali

**Affiliations:** 1 Obstetrics and Gynaecology, Jawaharlal Nehru Medical College, Datta Meghe Institute of Medical Sciences, Wardha, IND; 2 Anatomy, Jawaharlal Nehru Medical College, Datta Meghe Institute of Medical Sciences, Wardha, IND

**Keywords:** obesity, hormone levels, stress, irregular menstrual cycle, physical diagnosis, hyperandrogenism, genetics, hirsutism, young adolescents, s: polycystic ovary syndrome

## Abstract

Polycystic ovarian syndrome (PCOS) is a metabolic, reproductive, and psychological complex series of disorders that impacts a woman throughout her lifespan. PCOS is a disorder of hormonal imbalance occurring in women of reproductive age. This disorder is characterized by high levels of male androgens like testosterone. This can lead to symptoms like irregular periods, amenorrhea (absence of menstruation), anovulation (absence of ovulation), hirsutism, acne, and obesity. PCOS also causes metabolic impairment. Multiple peripherally arranged immature follicles of about 2-5mm in diameter are present in the ovary. These follicles do not mature due to hormonal imbalances leading to an irregular menstrual cycle. PCOS is a metabolic, reproductive, and psychological complex series of disorders that impacts a woman throughout her lifespan. Polycystic ovarian syndrome is not a fatal or life-threatening disorder as its main complication is infertility. PCOS can be a root cause of serious medical conditions like obesity, hypertension, type-2 diabetes mellitus due to insulin resistance, endometrial cancers, ovarian cancer, etc. Stress may cause the hormone levels in the pituitary to fluctuate. Since the menstrual cycle is hormone-based, there are apparent irregularities.

## Introduction and background

Polycystic ovarian disorder or polycystic ovarian syndrome (PCOS) is a widespread disorder with endocrine-metabolic impairment [1]. The pathophysiology of this disease is complex and shows the interactions of genetic alterations, primary ovarian disruptions, neuroendocrine changes, endocrine and metabolic predisposition, hyperinsulinemia, obesity, and resistance insulin resistance and adiponectin levels. The common symptoms seen in patients are acne, hirsutism, irregular menstrual cycle for two years since menarche, and hyperandrogenism. Fertility aspects and transition with adolescents must be handled [2]. The diagnostic criteria include chronic anovulation, high androgen levels, and polycystic ovaries depicted in the ultrasound [3]. There are three phenotypes of PCOS: (i) Hypersecretion of androgens, cysts in the ovary, occasional anovulation, (ii) Hypersecretion of androgens and occasional anovulation, and (iii) Hypersecretion of androgens and cysts in the ovary. PCOS can depict other complications like high sugar levels, obesity, metabolic malfunction, and difficulty in conceiving. These complications are a result of the malfunction of pituitary gland secretion, oogenesis, and insulin secretion. 

The investigations for PCOS include general physical examination, pelvic sonography, blood test for hormones, family history, and hirsutism [4]. Vitamins play a significant role in the various functions of the body. Vitamin B complex and vitamin C help in iron absorption. Chromium is a crucial element in insulin and sugar metabolism [5]. This disease usually is underdiagnosed or misunderstood with other disorders by endocrinologists [6]. The diagnosis of PCOS is controversial. Therefore, assessment and management are inconsistent. The need of female adolescents with PCOS are not being adequately met [7]. Drugs like metformin, oral contraceptives, and myoinositol for adolescents with PCOS can be helpful. Oral contraceptives prescribed to the patient are synthetic progesterone and combined progesterone-estrogen pills. Results show that there has been improvement in the regularity of the menstrual cycle, reduction in acne of the face, reduction of obesity, reduction in blood glucose level, and improved total cholesterol and low-density lipoprotein. However, treatment should only be continued if fewer side effects exist [8]. Before using oral contraceptives, lifestyle, dietary, and body mass index (BMI) changes should be made. Medical nutrition therapy and nutritional supplements have improved patients' signs and symptoms. This should be an ideal approach to restoring ovulation [9]. Hormonal contraceptives are the first line of management for menstrual irregularities, hirsutism, and acne. Treatment for infertility includes clomiphene. Lifestyle innervation is beneficial for obesity and other health benefits [10]. Thiazolidinediones have a good benefit ratio. Myoinositol efficiently brings about normal ovarian function and improves the fetus's ovum and quality [11]. The irregular menstrual cycle and other symptoms have normalized. It takes 4-12 months to improve the condition and relieve the symptoms. The resolution of cysts has also been seen in the ultrasound with homeopathic treatment. Cases were followed up with clinical and ultrasound evidence. There are minimum side effects as compared to conventional treatment [12].

Combined oral contraceptives are also effective treatments but have side effects for 16 weeks. Oral contraceptives cannot be given to teenagers for more than three years [13]. The nutritional studies showed that diet should be prioritized for the treatment of this disorder. Girls with PCOS met the acceptable macronutrient distribution ranges for carbohydrates, fats, and protein. However, no differences were recorded in the food or dietary quality intake between women with or without PCOS. A deficient and unique target for dietary interventions supports the position of new guidelines to incorporate healthy lifestyle modifications for treatment [14].

## Review

Methology

We undertook a systematic search through PubMed and CENTRAL in August 2022 using keywords such as "Polycystic Ovarian Syndrome" and "adolescence" (((Polycystic Ovarian Syndrome [Title/Abstract]) OR (PCOS [Title/Abstract])) OR ("polycystic ovarian syndrome" [MeSH Terms]) AND (("Adolescence" [Title/Abstract]) OR (QoL [Title/Abstract])) OR ("adolescence" [MeSH Terms]). We additionally searched for key references from bibliographies of the relevant studies. The search was updated in February 2022. A total of 353 articles were searched about the management of PCOS in adolescent girls in the age group of 12-18 years. Search strategies were putting terms like polycystic ovarian* add with or PCO*, and adding the Medical Subject Headings (MeSH) terms with the name PCOS. Filters for clinical trials, meta-analyses, randomized control trials, and systematic reviews were added. Additional filters for ages 13-18 years were added. One reviewer (AA) independently monitored the retrieved studies against the inclusion criteria, in the beginning, based on the title and abstract and then on full texts. Another reviewer (DGF) then reviewed approximately 20% of these studies to validate the inclusion of studies. Differences were resolved through discussion. 

Pathophysiology

It is imperative not to misinterpret typical puberty features with PCOS. It takes about two to three years for a teenager's menstrual cycle to come on track or become regular. The predisposing factors in childhood and puberty are shown in Table 1 [15].

**Table 1:** Table comparing PCOS and non-PCOS components PCOS: polycystic ovarian syndrome

	PCOS	Non-PCOS
Predisposing Factors	Genetic factors in utero programming	-
Childhood ( Pre-puberty)	Increased anti-Mullerian hormone levels	-
Puberty	Premature adrenarche, hyperandrogenism, Increased gonadotropin-releasing hormone, pulse frequency, bulky ovaries, irregular menses, hyperinsulinemia	Acne, increased ovarian volume, insulin resistance of puberty

**Figure 1:**
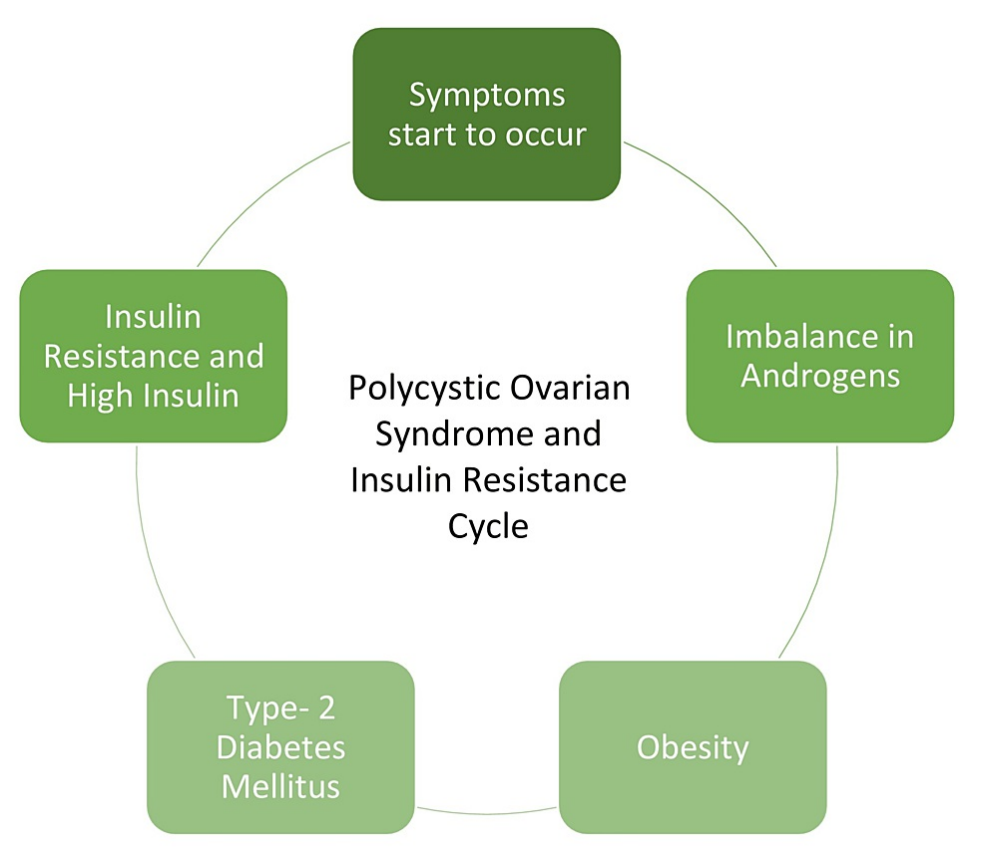
Link between diabetes and polycystic ovarian syndrome Image credit: Avanti Adone

There is a direct link between PCOS and insulin resistance. Females who suffer from PCOS are susceptible to type-2 diabetes mellitus. Figure 1 shows how PCOS leads to insulin resistance [16].

Etiology and pathophysiology

PCOS affects 5-10% of women of reproducible age, but its etiology and pathophysiology are still not quite understood. However, genetic factors and hypothalamic and ovarian dysfunction are the main reasons for all the symptoms. In adolescents, both the criteria of hyperandrogenism and irregular cycles are needed but ovarian morphology is not included due to poor specificity. The manifestations usually arise in childhood and then evolve into adolescence and reproductive age. The pathophysiology of this disorder was hypothesized to be based on interactions between the host, genetics, environment, and agent. Environmental factors and genetics could be a complex interplay [17]. Eating disorders like bulimia and recurrent dieting can lead to PCOS in adolescents. They can lead to the epigenetic dysregulation of the hypothalamic-pituitary-gonadal (HPG.) axis, thereby impacting oogenesis. Stress is a main related risk factor for PCOS, which can then lead to overeating, anxiety, and emotional distress. Doctors should consider the importance of psychological stress and eating disorders while dealing with these disorders [18]. Anti-Müllerian hormone (AMH) is elevated in this disorder, altering the ovulation with endocrine alterations and playing an inhibiting role in oogenesis and presenting to follicular arrest. AMH inhibitory action on follicle-stimulating hormone (FSH)-induced aromatase production likely presents hyperandrogenism in PCOS, further enhancing insulin resistance in these women [19]. Obesity in teenage girls might elevate the severity of the disease; therefore, it underlines the prime importance of early diagnosis and treatment. It may be advisable to define adolescent PCOS according to Rotterdam consensus criteria [20]. 

PCOS diagnosis in young females (teenagers) must be based on biochemical or clinical symptoms of hyperandrogenism and irregularity in the menstrual cycle. However, the diagnosis should be separated until at least two years following the beginning of the first menstrual cycle as these symptoms may also be associated with normal pubertal development. Until then, the main goal should be managing symptoms [21]. PCOS is a complex, multifaceted disorder and should be diagnosed and treated in pubescent girls after considering the patient's complete diagnostic picture, metabolic risks, and individual concerns to both avoid over-diagnosis and yet be able to provide early and meaningful treatment [22]. Abnormal uterine bleeding is the most routine gynaecological complaint of young girls. Patients often complain about heavy, prolonged menstrual bleeding. Many lethal diseases like hypothyroidism, hyperprolactinemia, and hyperandrogenism are possible causes of abnormal uterine bleeding. This can be brought in control by oral contraceptives and iron supplementation [23]. Although the cause of PCOS is unknown, it is known to be a heterogeneous disorder that might involve genetic and environmental factors contributing to its phenotypic expression.

An endocrine-disrupting chemical known as bisphenol A (BPA) causes PCOS. Increased bisphenol concentrations are seen in teenage and adult females with PCOS compared to reproductively healthy ones and are positively correlated with hyperandrogenemia, which shows the importance of chemicals [24]. Regular check-ups and close surveillance are necessary for every stage of life, like adolescence, reproductive age, and old age, to reduce complications and risks that could affect the patient's offspring as it is genetically transferred [25].

Complications

PCOS could be a cause for cessation of the menstrual cycle or primary or secondary amenorrhea. If it is accompanied by hirsutism and acne, laboratory tests should be done. Tests for hormones like FSH, luteinizing hormone, thyroid stimulating hormone, prolactin, androgen levels from the pituitary, and insulin from the pancreas must be measured. The priority should be the recovery of ovulation, menstrual cycle restoration, and the prevention of long-term complications [26]. There is a high chance of increased risks of depression, anxiety, obsessive-compulsive disorder (OCD), and somatization. Screening for these disorders may be warranted to allow early Intervention [27]. While diagnosing PCOS in adolescents, disorders that mimic PCOS should be eliminated. Young girls with PCOS should be regularly followed up for short-term and long-term complications, even in adulthood [28]. Patients with these disorders are at a high risk of cardiovascular diseases and metabolic disorders [29].

The most recent genetic investigation found many loci close to genes involved in metabolism, ovarian function, and gonadotropin production. Despite the challenges provided by the phenotypic and genetic variation among PCOS-afflicted females, research on one location, the *DENND1A* gene, sheds light on ovarian steroids' origins. It has long been understood that AMH contributes significantly to ovarian dysfunction. Recent animal studies show that AMH stimulates enhanced gonadotropin-releasing hormone and luteinizing hormone release, which link AMH to neuroendocrine instability [30]. Changes in lifestyle can help patients with PCOS who suffer from depression, a severe consequence. One hundred seventy-four females with PCOS were the subjects of a cross-sectional study, and data were gathered using sociodemographic, the Health Promoting Lifestyle Profile-2, and the Beck Depression Inventory-II questionnaires [31]. The factors of diet, interpersonal relationships, spiritual development, stress management, BMI, and the perceived stress of the illness lead to an aggravation of the symptoms. A change in lifestyle is required to lessen depression [31]. Because PCOS has relatively well-defined clinical, biochemical, and radiological markers in adult females, the symptoms in adolescents may overlap with those of normal puberty, making a diagnosis challenging. It is critical to distinguish between typical adolescence and real ovarian hyperandrogenism, both of which raise the risk of cardiovascular problems [32].

Adolescents should be concerned more with symptom management than fertility. Every young patient should receive individualized care [33]. Teenagers who are obese require increased attention from medical professionals and researchers, as well as beneficial early-stage interventions. Once the diagnosis of obesity has been made, it is vital to begin a holistic approach and show care for linked comorbidities as soon as feasible. Their weight condition substantially impacts the patient's pathophysiology [34]. The characteristic ovarian structure confirmed by ultrasound examination, elevated levels of luteinizing hormone, androgens, prolactin, and estradiol concentration with an upper limit are the distinguishing features of PCOS in addition to the clinical symptoms. Because of persistent anovulation and aberrant hormone balance brought on by the high basal prolactin level, the pituitary lactotrophs output was elevated [35]. This syndrome is way more complex than it looks. Adolescent patients are already under peer pressure and mental stress. They undergo many mood swings. Hence, handling such cases might be challenging [36]. PCOS can affect self-confidence and body image [37]. Teenagers have to cope with emotions and frustrations. Being stress-free is a critical aspect of treating this disorder. Communicating openly with friends and family can give the patient strength and a sense of support in dealing with the psychological and emotional components of the syndrome. Counselling and therapy help these patients in stress management and thereby in managing some of the few symptoms. PCOS is called a syndrome because the patient can experience many symptoms at the same time. Therefore, the management of symptoms and long-term complications in teenagers is essential. PCOS can affect mental health drastically as it indirectly influences body image [38].

When adolescent females receive lifestyle interventions like nutrition and exercise, their levels of luteinizing hormone and the free androgen index (FAI) significantly improve compared to baseline. Additionally, this research showed that dietary changes alone were linked to a considerable drop in BMI. Significant changes in menstruation, AMH, and triacylglycerol (TAG) levels were linked to exercise programs [39]. About 11-21% of women of reproductive age have PCOS and around 75% show polycystic ovaries on ultrasonography. Physicians must denote the patient's phenotype when concluding a diagnosis. It is a highly complex, multifactorial, and highly inherited polygenic disorder. Long-term risks include mood swings, psychosexual disorders, possible ovarian malignancy, endometrial atypia and carcinoma, cardiovascular and cerebrovascular disorders, high blood pressure, fertility and obstetrics complications, glucose intolerance, type-2 diabetes mellitus, hepatic stenosis, and metabolic malfunctions [40].

Discussion and prevention

A study was performed by the Australian Longitudinal Study of Women's Health comparing women with or without self-diagnosed PCOS. The primary outcome was that women reporting PCOS, when compared with women not reporting PCOS, had a higher prevalence of anxiety, depression, and stress. Even after adjusting to BMI, infertility, and sociodemographic factors, women with PCOS are still more likely to be anxious, depressed, and stressed. Many young females have PCOS but are unaware of it. Seventy percent of women are undiagnosed. The primary cause of PCOS involves a disturbance in insulin efficiency or low insulin levels since childhood. There are multiple secondary causes, such as environmental factors, socioeconomic status, poor and unhealthy nutrition, alcohol intake, and smoking of cigarettes. PCOS is quickly passed on genetically. A female suffering from PCOS could be subfertile and can genetically pass on this disorder, while the male partner could be fertile. A study shows that single nucleotide polymorphism is significantly related to the genetic risk of PCOS with association within three loci was depicted. Adolescents having PCOS do not need to be infertile in their reproductive age. This is because they ovulate spontaneously. However, the ovulation frequency is not certain, but a study reveals that women with PCOS ovulate 32% of their whole reproductive age. Pubic and axillary hair pattern examination must also be necessary during the diagnosis. PCOS may show a certain pattern of alopecia (hair loss). Alopecia could be a vital sign of hormonal disturbances, also known as androgenic alopecia [41].

One of the most rational treatment methods for PCOS is inositol, a dietary supplement with proven effect on ovulation without any adverse side effects if taken in recommended doses. Alternative treatment methods include insulin sensitizers, selective estrogen receptor modulators, and aromatase inhibitors. Among these, the aromatase inhibitor letrozole and the combination of clomiphene citrate and metformin have the highest rate of causing ovulation. Ovarian electrocautery and low-dose FSH stimulation are also some other viable options. PCOS has an essential impact on other metabolism taking place in the body. It vastly affects lipid metabolism. Therefore, the classical sign of PCOS in 50-60% of women is obesity. Obesity is associated with hyperlipidemia (hypertriglyceridemia and hypercholesteremia). PCOS causes a litany of diseases like infertility, insulin resistance, obesity, heart problems, and much more. It is a polygenic, multifactorial, inflammatory, autoimmune disease that occurs largely due to lifestyle error. Due to rapid advancements in biochemical tests and ultrasound imaging, PCOS can now be detected as early as possible. In recent times, a considerable amount of information has been garnered on PCOS, leading to the development of interventions like oral contraceptive pills and hormone therapy which can be used to reverse the effects of PCOS. However, lifestyle changes remain the primary correction therapy for controlling PCOS [42]. PCOS is the leading cause of female infertility worldwide and is linked to an increased risk of diseases like type-2 diabetes mellitus, psychiatric disorders, and gynaecological cancer. Despite a large number of cases, the main etiology of PCOS remains unknown. Although the genetic loci of PCOS account for only 10% of its heritability, male and female relatives of women suffering from PCOS are at high risk of developing PCOS-associated reproductive and metabolic disorders. Hyperandrogenism is the leading cause of PCOS [43].

PCOS is a lifestyle disorder often associated with weight gain and obesity, which increase the severity of PCOS. Hence, weight management includes modest weight loss, maintenance of weight, and prevention of excess gestational weight gain. Despite proper evidence-based guidelines, the complexity, intensity, and behavioural changes associated with lifestyle interventions are not adequately understood in PCOS. Throughout the world, behavioral theories, change patterns, and psychological correlations between weight management have been thoroughly explored in the general population, which includes both reproductive and perinatal women. The outcomes of all the interventions correlate to the behavioural and psychological strategies, including self-monitoring, cognitive restructuring, and relapse prevention.

## Conclusions

Teenagers go through extreme amounts of emotional changes during puberty. This leads to stress and anxiety, especially in young females who show rapid growth. Being stress-free is an important aspect of the treatment of this disorder. Communicating openly with friends and family can give the patient strength and a sense of support in dealing with the psychological and emotional components of the syndrome. The management of symptoms and long-term complications in teenagers is essential. Counseling and therapy help in stress management and thereby in managing some of the few symptoms. The recognition of signs and symptoms of PCOS even before the onset of adolescence is essential.

Various types of treatment can manage the symptoms but this condition is not curable. It is a chronic disorder that can last for many years or a whole reproductive age. There should be thorough diagnosis through ultrasonography, blood tests for hormones, complete physical and psychological evaluation and examination, and frequent check-ups. There could be a stigma around females for having lots of facial hair due to excess male hormones, trouble losing weight, and acne. There is hair loss and pelvic pain. Teenagers are at high risk if they have an unhealthy lifestyle. Thus, PCOS is a common condition that can have long-term complications if left undiagnosed and untreated. Early diagnosis and quick treatment can improve the life quality and fertility of adolescent females.
